# Lessons learned from contemporary glioblastoma randomized clinical trials through systematic review and network meta-analysis: part 2 recurrent glioblastoma

**DOI:** 10.1093/noajnl/vdab029

**Published:** 2021-02-12

**Authors:** Shervin Taslimi, Vincent C Ye, Patrick Y Wen, Gelareh Zadeh

**Affiliations:** 1 Division of Neurosurgery, Department of Surgery, University of Toronto, Toronto, Ontario, Canada; 2 Center for Neuro-Oncology, Dana-Farber Cancer Institute, Boston, Massachusetts, USA

**Keywords:** glioblastoma, network meta-analysis, randomized control trials, recurrent, systematic review

## Abstract

**Background:**

There exists no consensus standard of treatment for patients with recurrent glioblastoma (GB). Here we used a network meta-analysis on treatments from randomized control trials (RCTs) to assess the effect on overall survival (OS) and progression-free survival (PFS) to determine if any consensus treatment can be determined for recurrent GB.

**Methods:**

We included all recurrent GB RCTs with at least 20 patients in each arm, and for whom patients underwent standard of care at the time of their GB initial diagnosis. Our primary outcome was OS, with secondary outcomes including PFS and adverse reactions. Hazard ratio (HR) and its 95% confidence interval (CI) of the comparison of study arms regarding OS and PFS were extracted from each paper. For comparative efficacy analysis, we utilized a frequentist network meta-analysis, an extension of the classic pair-wise meta-analysis. We followed the Preferred Reporting Items for Systematic Reviews and Meta-analyses.

**Results:**

Fifteen studies were included representing 29 separate treatment arms and 2194 patients. In our network meta-analysis, combination treatment with tumor-treating field and Vascular endothelial growth factor (VEGF) inhibitor ranked first in improving OS (*P* = .80). Concomitant anti-VEGF and Lomustine treatment was superior to Lomustine alone for extending PFS (HR 0.57, 95% CI 0.41–0.79) and ranked first in improving PFS compared to other included treatments (*P* = .86).

**Conclusions:**

Our analysis highlights the numerous studies performed on recurrent GB, with no proven consensus treatment that is superior to the current SOC. Intertrial heterogeneity precludes drawing strong conclusions, and confidence analysis was low to very low. Further confirmation by future trials is recommended for our exploratory results.

**Key Points**

- Our analysis highlights the numerous studies performed on recurrent glioblastoma, with no proven consensus treatment.
- Intertrial heterogeneity precludes drawing strong conclusions, and confidence analysis was low. This raises the need for more consistent standardization across trials.

**Importance of the Study**

To survey the current clinical landscape in the treatment of recurrent glioblastoma, we performed a systematic review and network meta-analysis of randomized control trials to assess the positive impact on overall survival and progression-free survival. Our analysis highlights the numerous studies performed on recurrent glioblastoma, with no proven consensus treatment that is superior. Intertrial heterogeneity precludes drawing strong conclusions, and confidence analysis was low to very low. Further confirmation by future trials is recommended for our exploratory results.

Glioblastoma (GB), is the most common and deadliest primary brain tumor in adults.^1^ There does not yet exist a curative treatment for GB.^2^ The current standard of care treatment protocol for newly diagnosed GB is a well-established protocol involving concomitant radiotherapy and temozolomide (TMZ), followed by adjuvant TMZ.^3^ The introduction of this paradigm has had a tangible impact on overall survival (OS) and progression-free survival (PFS). Inevitably, the disease recurs with an overall 2-year survival rate less than 30%. Contrary to the established treatments for newly diagnosed GB, there is considerable variability and controversy when it comes to the best treatment options for recurrent GB. Presently, only approximately 30% of recurrent GBs undergo a second surgical resection.^4^ There is no consensus best treatment protocol for recurrent GB.

It is thought that once a tumor recurs, it is less sensitive to therapies received during the first round of treatment.^5,6^ This may be the result of selective pressures on the immune system or changes in the tumor microenvironment that exacerbates genetic and epigenetic heterogeneity within the tumor, including mutations in mismatch repair genes.^7,8^ Treatment regimens for recurrent GBs include alkylating agents such as TMZ or lomustine, bevacizumab, reirradiation, or experimental therapies.^2^

In order to assess evidence-based clinical outcomes, and to provide a summative overview of recurrent GB therapies, we performed a systematic review and network meta-analysis comparing the efficacy of differential treatment regimens in phase 2 and 3 randomized control trials (RCTs) for recurrent GB. Network meta-analyses have been used in neurosurgery recently to assess the optimal treatment regimens for elderly patients with GB.^9–11^ Traditional meta-analysis offers comparisons between 2 treatment arms; the use of a network meta-analysis was ideal for our scenario in which multiple different treatment regimens were compared using both direct head-to-head comparisons of interventions within various trials and indirect comparisons across different trials based on a common control comparator.

## Methods

### Literature Search and Systematic Review

We conducted our systematic review and network meta-analysis based on a predefined protocol in accordance with the Preferred Reporting Items for Systematic Reviews and Meta-Analyses Extension statement for reporting on network meta-analyses.^12^ Databases including MEDLINE (PubMed and Ovid), Embase, and Web of Science were searched through July 1, 2019. We used, in relevant combinations, keywords and MeSH (Medical Subject Heading) terms pertaining to the patient population disease (high-grade glioma, GB) and clinical trial. Abstracts were screened for potential inclusion, and full-text articles were reviewed for articles of interest.

Inclusion criteria included randomized control clinical trial of phase 2 or 3 trials with 20 or more patients in each treatment arm, patients with recurrent GB (astrocytoma grade IV) having undergone maximal safe resection, and concomitant chemoradiotherapy and adjuvant chemotherapy as first-line treatment for the initial disease. All treatments during recurrence were eligible. Articles were excluded if results on GB patients could not be separated from non-GB patients included in trials and if data on primary and secondary outcomes were not available.

Our primary outcome was OS from pooled outcomes of RCTs (hazard ratio [HR]), with the secondary outcome being PFS (HR) and side effects of treatments. Studies in which data on the primary and secondary outcomes could not be extracted or could not be obtained from a corresponding author were not included in the analysis. Other demographic and clinical factors collected included the number of patients, age, baseline status, Isocitrate dehydrogenase (IDH) and O[6]-methylguanine-DNA methyltransferase (MGMT) methylation status, previous treatments, surgical treatments and extent of resection (EOR), performance status, and side effects.

Quality assessment of the included studies was done using Cochrane’s Risk of Bias Tool for randomized trials.^13^ This previously validated tool is designed to assess the quality and risk of bias for randomized controlled trials.

### Statistical Analysis

A network meta-analysis was utilized to simultaneously compare the efficacy of multiple different treatments across studies. This approach synthesizes metrics of both direct and indirect comparisons to refine and generate estimates of all possible pair-wise comparisons within a network.^14–16^ Treatments that did not form pairs or that do not map onto the network cannot be included in the network meta-analysis We made an estimate of treatment effect via direct comparisons between treatment groups within a single trial and an indirect comparison of treatment effect between different trials with a common comparator. When both direct and indirect evidence of comparison between treatment modalities were available, the treatment effect was synthesized together to yield a network treatment effect. We then ranked the treatments according to the probability of each treatment being the most effective based on Rücker and Schwarzer method.^17^ We assessed heterogeneity using Cochran’s Q statistics where a *P* value of .1 was considered significant heterogeneity. We used a random effect model if the data recognized as heterogenous, otherwise, a fix effect model was used. A two-way *P* value of less than .05 was considered statistically significant for all analyses. R software version R 3.6.3 was used for all analyses.

To assess the confidence in the results of the network meta-analysis, we utilized a previously described method, the Confidence in Network Meta-Analysis (CINeMA) framework and software.^18,19^ This framework incorporates 6 domains to determine the level of confidence in the network meta-analysis results: (1) within-study bias, (2) reporting bias, (3) indirectness, (4) imprecision, (5) heterogeneity, and (6) incoherence.

### Special Considerations for Analysis

Vascular endothelial growth factor (VEGF) inhibitors have been shown to decrease vascular permeability and cerebral edema.^20,21^ When assessing PFS, it is important to clarify the definitions used, as studies may have disproportionate outcomes depending on if they factor in T2 FLAIR signal or not, particularly in the setting of VEGF inhibitor use. We therefore planned a sensitivity analysis of PFS by including only studies that used the Response Assessment in Neuro-Oncology (RANO) criteria^22^ for assessing the progression of the disease and excluding the studies that used the older Macdonald criteria.^23^

## Results

**Figure 1.**
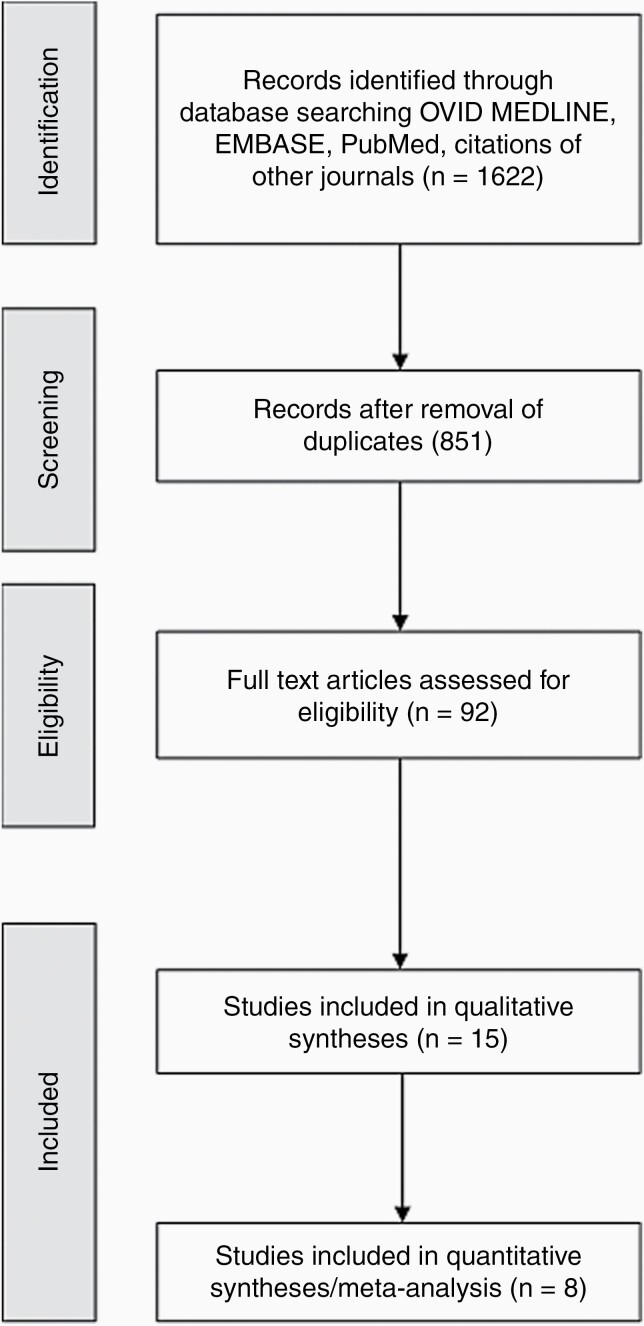
Study flow diagram.

Our literature search included 1622 initial results. After removal of duplicates and abstract screening, 92 papers underwent full-text review. Fifteen studies were included representing 29 separate treatment arms (Figure 1). A total of 2194 patients with recurrent GB were included. Study characteristics are summarized in Table 1.^5,24–40^ In the entire study population, the reported prevalence of MGMT promoter methylation was 16.9%, and the presence of IDH mutation was 2.7%. EOR was not robustly reported. Among the 4 studies that did report EOR, gross total resection was achieved in 55% at initial diagnosis, subtotal resection in 30.3%, and biopsy only in 14.5%.^28,30,31,33^ For the entire cohort, 19.5% of patients underwent redo surgery for their recurrent disease. The majority of patients included in these trials had a good performance status with a Karnofsky Performance Status (KPS) ≥70 or an ECOG/WHO status ≤2: Batchelor et al.^24^ included one patient each in the lomustine alone arm and the lomustine/cediranib arm with KPS <70, Field et al.^31^ reported 11 patients in the bevacizumab/carboplatin arm and 12 patients in the bevacizumab monotherapy arm with KPS <70, Duerinck et al.^30^ included 12 patients in the axitinib arm with ECOG >2, while Narita et al.^35^ reported 8 patients with ECOG >2 in the treatment arm and 2 patients in the placebo arm with ECOG >2.

**Table 1.** Summary of Included Studies

Study	Treatment	No. Patients	Age	PFS Criteria	Mechanism	% MGMT Methylated	% IDH Mutant	Redo Surgery (%)	Median OS (months)	OS HR	Median PFS (months)	PFS HR
Batchelor (2012)	Lomustine	65	54	Other	Alkylating nitrosourea	NR	NR	36.9	9.8	NR	2.73	
	Cediranib	129	54		Anti-VEGF	NR	NR	38.2	8	1.43	3.07	1.05
	Cediranib/Lomustine	131	54		Anti-VEGF/alkylating nitrosourea	NR	NR	38.0	9.4	1.15	4.17	0.76
Bogdahn (2011)	10 μg Trabedersen	40	46.5	NR	TGF-β	NR	NR	NR	7.3	—	NR	—
	80 μg Trabedersen	49	44.0		TGF-β	NR	NR	NR	10.9	—	NR	—
	TMZ/PCV	45	45.0		Alkylation	NR	NR	NR	10	—	NR	—
Brandes (2016)	Galunisertib/Lomustine	79	57.5	NR	TGF-β/alkylating nitrosourea	NR	38.0	NR	6.7	1.13	1.8	—
	Galunisertib	39	56.6		TGF-β	NR	10.5	NR	8	0.93	1.8	—
	Lomustine	40	56.9		Alkylating nitrosourea	NR	2.5	NR	7.5	—	1.9	—
Brown (2016)	Cediranib	19	61.0	RANO	Anti-VEGF	NR	NR	21.0	5.5	—	2.8	
	Cediranib/Gefitinib	19	55.0		Anti-VEGF	NR	NR	10.0	7.2	0.68	3.6	0.72
Cloughesy (2017)	Onartuzumab/Bevacizumab	91	57.0	RANO	Anti-MET/anti-VEGF	37.5	6.9	NR	8.8	1.45	3.9	1.06
	Bevacizumab	53	55.0		Anti-VEGF	48.1	8.8	NR	12.6	—	2.9	—
Cloughesy (2019)	Neoadjuvant Pembrolizumab	16	55.4	RANO	PD1 inhibitor	38.0	19.0	100	13.7	0.39	3.3	0.43
	Adjuvant Pembrolizumab	16	59.3		PD1 inhibitor	69.0	13.0	100	7.5	—	2.4	—
Duerinck (2018)	Axitinib	50	55.0	RANO	Anti-VEGF	33.0	NR	41.0	7.25	—	12.4	
	Axitinib/Lomustine	29	56.0		Anti-VEGF/alkylating nitrosourea	25.0	NR	55.0	6.875	—	13	0.58
Field (2015)	Bevacizumab	62	55.0	RANO	Anti-VEGF	NR	NR	50.0	7.5	—	3.5	—
	Bevacizumab/Carboplatin	60	55.0		Anti-VEGF/platinum	NR	NR	38.0	6.9	1.18	3.5	0.92
Lombardi (2019)	Lomustine	60	54.8	RANO	Alkylkating nitrosourea	46.0	0.0	23.0	5.6	—	1.9	—
	Regorafenib	59	58.9		Anti-VEGF	49.0	5.0	22.0	7.4	0.5	2.0	0.65
Narita (2019)	Personal Peptide Vaccine	58	52.5	NR	Peptide vaccine	NR	NR	NR	8.4	1.13	NR	—
	Placebo	30	59.0		—	NR	NR	NR	8.0	—	NR	—
Reardon (2015)	Afatinib	41	56.6	RANO	Anti-VEGF	NR	NR	NR	9.8	—	0.99	
	Afatinib + TMZ	39	55.4		Anti-VEGF/alkylation	NR	NR	NR	8	—	1.53	
	TMZ	39	56.9		Alkylation	NR	NR	NR	10.6	—	1.87	
Stupp (2012)	TTF alone	120	54.0	Macdonald	Anti-mitotic	NR	NR	25.0	NR	0.86	2.2	0.81
	“Active chemo”	117	54.0		—	NR	NR	28.0	NR	—	2.1	
Weathers (2016)	Bevacizumab	36	—	NR	Anti-VEGF	NR	NR	NR	8.3	—	4.11	0.71
	Bevacizumab/Lomustine	35	—		Anti-VEGF/alkylating nitrosourea	NR	NR	NR	9.6	—	4.34	
Wick (2017)	Lomustine	149	59.8	RANO	alkylating nitrosourea	24.8	NR	18.8	8.6	0.95	1.5	
	Bevacizumab/Lomustine	288	57.1		Anti-VEGF/nitrosourea	23.3	NR	20.5	9.1	—	4.2	0.49
Wick (2014)	Radiotherapy	30	59.0	Macdonald	RT	57.0	0.0	NR	11.5	0.6	2.5	
	Radiotherapy + APG101	61	57.1		RT/CD95 ligand	70.0	10.0	NR	11.5	—	4.5	0.49
Kesari (2017)	“Second-Line Chemo”	60	58	—	—	23.0	NR	NR	9.2	—	—	—
	“Second-Line Chemo” + TTF	144	57		Anti-mitotic	24.0	NR	NR	11.8	0.695	—	—
	Bevacizumab	26	NR		Anti-VEGF	NR	NR	NR	9	—	—	—
	Bevacizumab + TTF	61	NR		Anti-VEGF/anti-mitotic	NR	NR	NR	11.8	0.606	—	—

NR, not reported.

Anti-VEGF therapies were the most common agents in these trials, while other common treatments included anti-TGF β, alkylating nitrosourea, and anti-PD1. Two studies looked at tumor-treating fields (TTFs) with or without best second-line chemotherapy, left up to the discretion of the treating physician. There were 1383 patients in treatment arms, as compared to 811 patients in control arms. Control treatments varied between studies, likely representative of the lack of consensus treatment for recurrent GB. The prevalence of MGMT promoter methylation in the treatment arms was 13.5%, as compared with 23.4% in control arms. To strengthen the network connection and allow for an increased number of direct and indirect comparisons, VEGF inhibitors were grouped.

### Quality of Evidence

The overall risk of bias based on the Cochrane Collaboration tool was low for all included studies. Detailed quality assessment results are available in Supplementary Figure 1.

### Survival Outcomes

Eight studies with sufficient survival outcomes data were included in our analysis, with 10 direct comparisons. This accounted for a total of 1784 patients; 387 received mono-anti-VEGF therapy, while 729 patients received anti-VEGF therapy in combination with another treatment. The average age of patients in this analysis was 56.2 years, with MGMT promoter methylation present in 29.7% of patients. Repeat surgical resection occurred in 28.1%.

**Figure 2.**
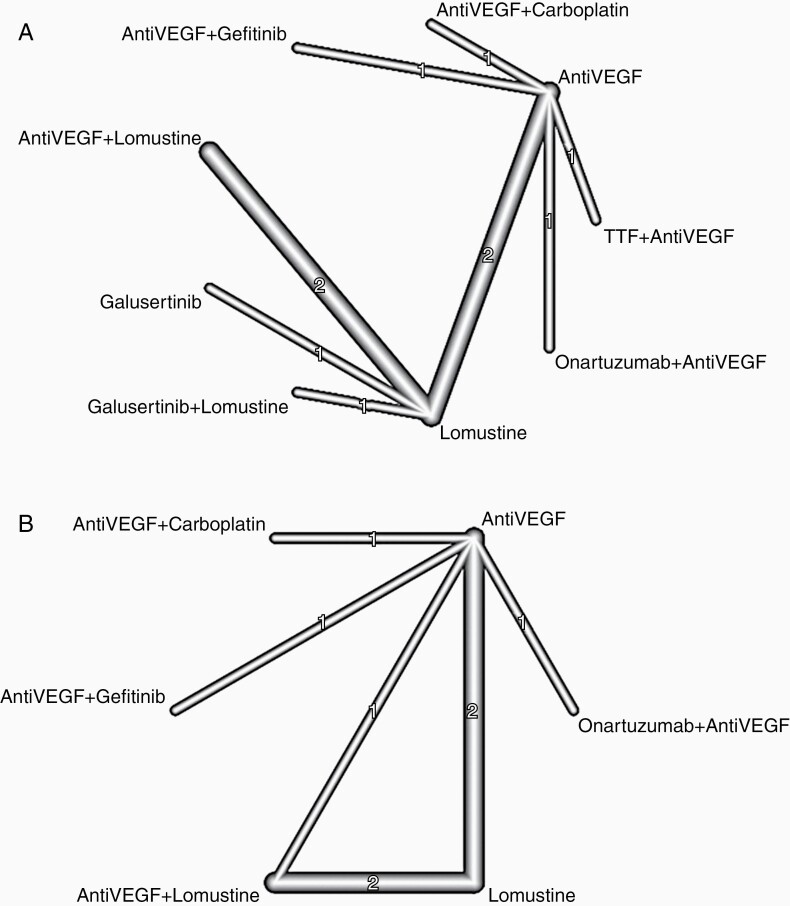
Node network graphs for (A) overall survival and (B) progression-free survival.

**Figure 3.**
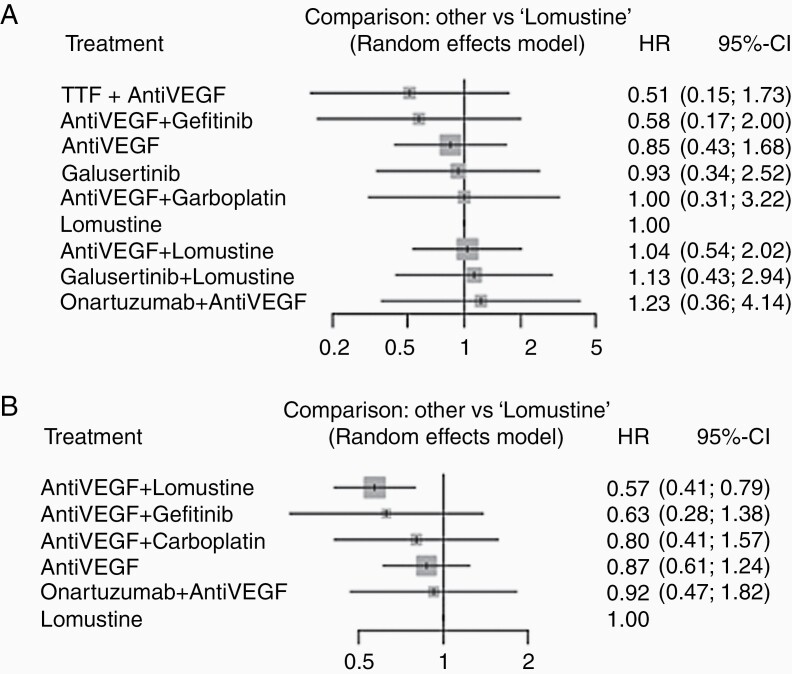
Forest plots for studies included in the meta-analysis for (A) overall survival and (B) progression-free survival.

**Figure 4.**
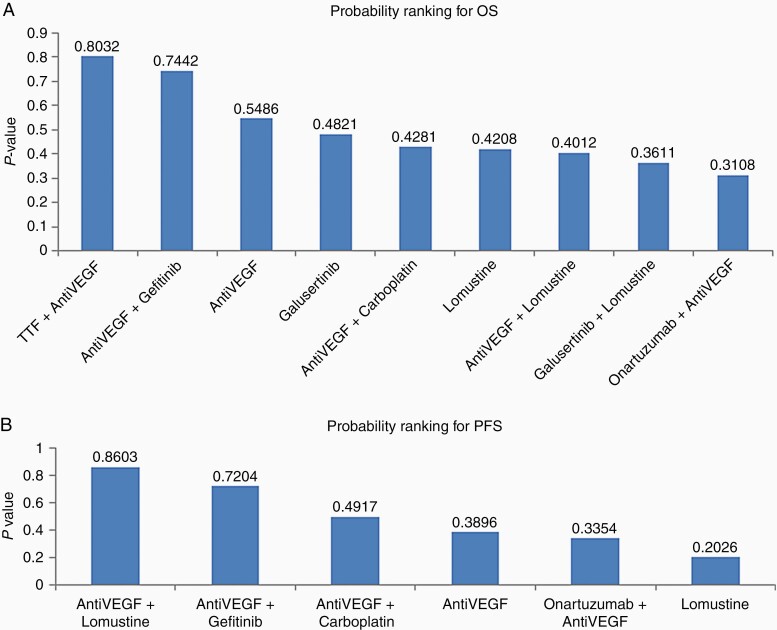
Treatment probability rankings for (A) overall survival and (B) progression-free survival.

The following treatments were included in the analysis: cediranib,^24,27^ galusertinib,^41^ geftinib,^27^ lomustine,^24,34,39,41^ onartuzumab,^28^ bevacizumab,^28,31,39^ carboplatin,^31^ TTF,^33^ and regorafenib.^34^ Treatments targeting VEGF pathways (bevacizumab, cediranib, and regorafenib) were combined into a single label “Anti-VEGF.” The network graph for OS meta-analysis is depicted in Figure 2A. Q statistics were significant (*Q* = 13.76, df = 2, *P* = .001) and as a result, we used a random effect model to pool the data (Supplementary Figures 2 and 3). Combination treatment with TTF and VEGF inhibitor had the greatest impact on OS when compared to Lomustine-only therapies (HR = 0.51, 95% confidence interval [CI] 0.15–0.73, Figure 3A). The probability ranking of these treatments showed that the combination of TTF and VEGF inhibitor had the highest probability of being the best treatment (*P* = .803, Figure 4A).

There was one study on TTF that could not be included in the meta-analysis since it did not overlap with other treatment arms. The 2 studies^33,36^ compared TTF with or without active second-line chemotherapy, against active chemotherapy alone. There were 120 patients who received TTF alone, 144 who received TTF in addition to active chemotherapy, and 177 patients who received chemotherapy alone. In these studies, TTF showed benefit on OS when used alone (HR = 0.86, 95% CI 0.66–1.2) or with active chemotherapy (HR = 0.695, *P* = .05).

A study on HLA personal peptide vaccine^35^ was similarly not included due to lack of overlap with other treatment arms—this study had a nonsignificant improvement in OS when compared with placebo (median OS 8.4 vs 8.0, HR 1.13, 95% CI 0.6–1.9, *P* = .621). Other studies that were excluded were reirradiation with or without APG101, a CD95 ligand (CD95L)-binding fusion protein,^40^ low-dose bevacizumab/Lomustine,^38^ axitinib,^30^ afatinib,^5^ and trabedersen^25^ (missing HR data).

Confidence analysis for OS was rated as very low for all comparison arms (Supplementary Figure 4).

### Progression-Free Survival

Seven studies with sufficient data were included for use in the meta-analysis, with 8 direct comparisons between treatments. Modalities included cediranib,^24,27^ lomustine,^24,30,34,39^ gefitinib,^27^ bevacizumab,^28,31,39^ onartuzumab,^28^ carboplatin,^31^ axitinib,^30^ and regorafenib.^34^ There were a total of 1264 patients included in the analysis with an average age of 56.2 years. The number of patients who received anti-VEGF therapy alone was 372, while 618 patients received another therapy in addition to anti-VEGF therapy. MGMT promoter methylation was present in 31.3% of patients in this analysis. Repeat surgical resection was performed in 29.7% of patients. RANO criteria were used to define PFS, except in one study.^24^

To strengthen the network connections and comparisons, treatments targeting the VEGF pathway (bevacizumab, cediranib, axitinib, regorafenib) were combined under the label of “anti-VEGF.” The network graph for PFS is shown in Figure 2B. Q statistics were significant (*Q* = 7.77, df = 3, *P* = .05) (Supplementary Figures 6 and 7). As a result, we used a random effect model to pool the data. Concomitant anti-VEGF and lomustine treatment was superior to lomustine alone for extending PFS (HR 0.57, 95% CI 0.41–0.79, Figure 3B). A concomitant anti-VEGF and gefitinib treatment was marginally better compared with lomustine alone in improving PFS (HR 0.63, 95% CI 0.28–1.38). The probability ranking of these treatments showed that, among included studies, concomitant anti-VEGF and lomustine treatment regimen was most likely to have an impact on PFS (*P* = .86), followed by anti-VEGF plus Gefitinib (*P* = .72, Figure 4B). The sensitivity analysis, by excluding the studies that did not define PFS based on RANO criteria, did not change the result (Supplementary Figure 5).

Reirradiation with or without APG101, a CD95 ligand (CD95L)-binding fusion protein,^40^ and afatinib^42^ were not able to be included in the network meta-analysis due to lack of treatment overlap.

Confidence analysis was rated as low for all comparisons for PFS (Supplementary Figure 8).

### Safety Analysis

Pooled analysis of adverse events (AEs) was not possible as a result of the heterogeneity within the reported variables. The frequency of AEs per patient in studies that reported AEs is given in Supplementary Table 1. In general, more AEs occurred in trial arms combining multiple therapeutic agents/modalities. Cediranib/gefitinib had the highest frequency of grade 3 or 4 AEs with 2.53 events per patient. TTF resulted in 1.32 grade 3 or 4 AEs per patient, as compared to 0.85 AEs per patient in the study control arm of active chemotherapy. The overall incidence of grade 5 AEs was extremely low.

## Discussion

GB continues to be one of the most malignant and resistant diseases to treat in oncology. While treatment for initial GB centers around radiotherapy and an alkylating chemotherapy drug, TMZ, multiple different agents have been studied for recurrent disease including therapeutics targeting VEGF, cell checkpoint pathways, other alkylating cancer agents, and anti-mitotic treatments; despite ample preclinical and clinical research, effective treatments do not yet exist for tumor recurrence. We performed a network meta-analysis of RCTs in the current literature to summate clinical evidence to date and to determine the efficacy of treatments in patients with recurrent GB.

Our analysis depicts the wide range of treatment modalities that have been studied in the treatment of GB and highlights the lack of a proven, consensus treatment. Our meta-analysis showed that concomitant anti-VEGF and lomustine treatment was superior to other treatments in improving PFS and TTF plus VEGF treatment was superior to all other included treatments in improving OS in patients with recurrent GB. Of note, we were not able to compare the effect of TTF with other treatments (eg, non-anti-VEGF combinations) in our meta-analysis due to the lack of overlap between study arms and the rest of the studies precluding inclusion within the network. Data from Stupp et al.^36^ comparing TTF with a wide range of chemotherapy agents did not show statistical significance for improving either PFS or OS, but showed a trend toward improved outcomes. However, Kesari et al.^33^ showed significant improvement of OS when TTF was used in addition to chemotherapy. Furthermore, it is important to note that the results from this trial must be viewed through the constraints and confounders that may arise from its post hoc nature. This trial was designed as a randomized trial of TTF for newly diagnosed GB, but a post hoc analysis was conducted on the recurrent disease. There was significant heterogeneity of first-line therapy received by the patients included in the post hoc analysis—including a subset of patients who had received TTF as first-line therapy and then continued on this treatment at the time of recurrence.

Taken together, given the heterogeneity of GB, combination therapies may be superior to monotherapy for the treatment of recurrent GB. The prevalence of certain mutations at the time of recurrence may play a role in deciding which combination therapy is most effective. However, combining therapies may subject a patient to more treatment side effects. It is important to view the results of our analysis bearing in mind its methodological intentions and limitations. The treatments identified via a network meta-analysis may not be the objective best treatment, but rather is determined to be the best treatment based on the included studies. For our analyses, we combined treatment arms with similar pathway targets, most notably anti-VEGF treatments. The different therapeutic agents, while all targeting VEGF, have differing biochemical and pharmacokinetic/pharmacodynamic profiles, as well as having effects on other molecular targets (eg, cediranib,^43^ regorafenib^44^). It may be that certain classes of anti-VEGF agents are in fact more efficacious (such as having increased blood–brain barrier permeability), but we were not able to determine this from our analysis. Further research and trials comparing different VEGF pathway therapeutics may be indicated. Using a CINeMA framework for assessing confidence in the results of a network meta-analysis,^18,19^ the confidence of the network meta-analysis scored low or very low. This again highlights the exploratory nature of our study and the need for future trials to elucidate future directions in the contemporary treatment of recurrent GB. Trials with a wide range of enrollment were included to allow adequate studies in the meta-analysis. One might expect that smaller trials will contribute more to intertrial heterogeneity and may negatively affect the confidence of the outcomes in these smaller studies. A network meta-analysis may have different study outcomes in the future with new, large trials and more robust inclusion criteria.

While upfront maximal safe cytoreduction is the standard of care in primary GB, the role of surgery for recurrent disease is much less clear and remains controversial. There is a well-established correlation between EOR and survival outcome for primary resection of tumours^45^–this correlation is substantially less robust in recurrent disease. The percentage of patients in the studies included in our literature review who underwent surgery ranged from 10% to 100%, although the majority of the studies had less than 40% of patients undergo redo surgery. EOR was not robustly reported in these studies, but of the studies which did report EOR, more than 80% of patients received either gross total or subtotal resection at the time of diagnosis.

There are several genetic markers in GB that portend important prognostic value. The most impactful are MGMT methylation and *IDH* mutation. While their impact on prognosis at disease recurrence is not as profound as at the time of initial diagnosis,^46^ they undoubtedly still contribute to disease course. MGMT methylation, in particular, is a prognostic marker of response to TMZ—at recurrence, evolutionary pressures from treatment and further tumor mutations^46,47^ may cause significant changes in the disease biology separate from its initial genotypic profile. The genetic data included in our listed studies were surprisingly quite limited and we were not able to assess the differential impact of treatments on patient subgroups with these mutations. As such, the results of our study may not be universally generalizable across all patient subgroups. Further research may elucidate which patients benefit most from treatment at recurrence.

## Conclusions

We present the first study using a network meta-analysis to examine RCT data on recurrent GB. Our analysis depicts the breadth of research on this topic and highlights the lack of consensus, proven treatment. Given the heterogeneity of the disease, it appears that combination treatments may be more effective than monotherapy alone. Further studies are required to elucidate specific treatment regimens and to study different subgroups of patients with recurrent GB.

## Supplementary Material

vdab029_suppl_Supplementary_Materials

## Notes

**Authorship Statement.** Conception and design: S.T., V.Y., and G.Z; development of methodology: S.T., V.Y., and G.Z.; acquisition of data (provided animals, acquired and managed patients, provided facilities, etc.): S.T., V.Y., and G.Z.; analysis and interpretation of data (eg, statistical analysis, biostatistics, computational analysis): S.T. and G.Z.; writing, review, and/or revision of the manuscript: S.T., V.Y., P.W., and G.Z.; administrative, technical, or material support (ie, reporting or organizing data, constructing databases): S.T. and V.Y.; study supervision: G.Z.
