# Supplementary material for: Lessons learned from contemporary glioblastoma randomized clinical trials through systematic review and network meta-analysis: part 2 recurrent glioblastoma
Source: Neurooncol Adv. 2021 Feb 12;3(1):vdab029. doi: 10.1093/noajnl/vdab029 (PMC8134527; doi:10.1093/noajnl/vdab029)
Supplement: vdab029_suppl_Supplementary_Materials [file vdab029_suppl_supplementary_materials.docx]

Supplemental Figure 1. Cochrane Review of Basis Summary for included studies


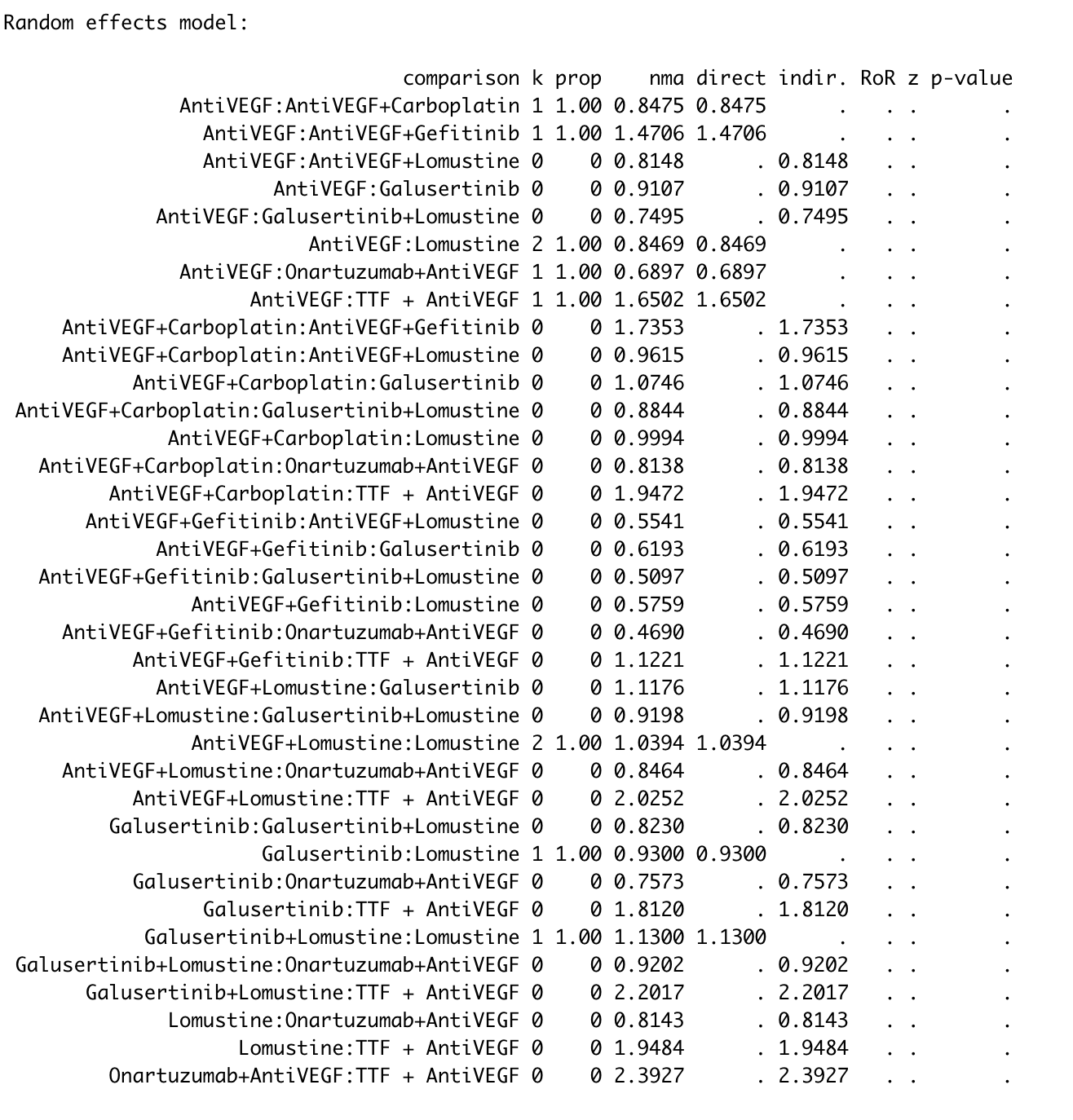


Supplemental Figure 2. Random effects model used in the meta-analysis of OS

| Treatment | AntiVEGF | AntiVEGF + Carboplatin | AntiVEGF + Gefitinib | AntiVEGF + Lomustine | Galusertinib | Galusertinib + Lomustine | Lomsutine | Onartuzumab + AntiVEGF | TTF + AntiVEGF |
| --- | --- | --- | --- | --- | --- | --- | --- | --- | --- |
| AntiVEGF | - | 0.85 (0.33 – 2.19) | 1.47 (0.52 – 4.17) | 0.81 (0.31 – 2.11) | 0.91 (0.27 – 3.05) | 0.75 (0.23 – 2.43) | 0.85 (0.43 – 1.68) | 0.69 (0.25 – 1.89) | 1.65 (0.61 – 4.50) |
| AntiVEGF + Carboplatin | 1.18 (0.46 – 3.05) | - | 1.74 (0.42 – 7.10) | 0.96 (0.25 – 3.69) | 1.07 (0.23 – 4.99) | 0.88 (0.20 – 4.00) | 1.00 (0.31 – 3.22) | 0.81 (0.20 – 3.24) | 1.95 (0.49 – 7.74) |
| AntiVEGF + Gefitinib | 0.68 (0.24 – 1.92) | 0.58 (0.14 – 2.36) | - | 0.55 (0.14 – 2.27) | 0.62 (0.13 – 3.05) | 0.51 (0.11 – 2.45) | 0.58 (0.17 – 2.00) | 0.47 (0.11 – 2.00) | 1.12 (0.26 – 4.76) |
| AntiVEGF + Lomustine | 1.23 (0.47 – 3.18) | 1.04 (0.27 – 3.99) | 1.80 (0.44 – 7.40) | - | 1.12 (0.34 – 3.70) | 0.92 (0.29 – 2.94) | 1.04 (0.54 – 2.02) | 0.85 (0.21 – 3.38) | 2.03 (0.51 – 8.07) |
| Galusertinib | 1.10 (0.33 – 3.68) | 0.93 (0.20 – 4.32) | 1.61 (0.33 – 7.96) | 0.89 (0.27 – 2.96) | - | 0.82 (0.21 – 3.28) | 0.93 (0.34 – 2.52) | 0.76 (0.16 – 3.65) | 1.81 (0.38 – 8.71) |
| Galusertinib + Lomustine | 1.33 (0.41 – 4.32) | 1.13 (0.25 – 5.12) | 1.96 (0.41 – 9.43) | 1.09 (0.34 – 3.48) | 1.22 (0.31 – 4.83) | - | 1.13 (0.43 – 2.94) | 0.92 (0.20 - 4.32) | 2.20 (0.47 – 10.3) |
| Lomustine | 1.18 (0.60 – 2.34) | 1.00 (0.31 – 3.22) | 1.74 (0.50 – 6.03) | 0.96 (0.50 – 1.87) | 1.08 (0.40 – 2.91) | 0.89 (0.34 – 2.30) | - | 0.81 (0.24 – 2.75) | 1.95 (0.58 – 6.56) |
| Onartuzumab + AntiVEGF | 1.45 (0.22 – 1.65) | 1.23 (0.31 – 4.90) | 2.13 (0.50 – 9.07) | 1.18 (0.30 – 4.72) | 1.32 (0.27 – 6.36) | 1.09 (0.23 – 5.10) | 1.23 (0.36 – 4.14) | - | 2.39 (0.58 – 9.90) |
| TTF + AntiVEGF | 0.60 ( | 0.51 (0.13 – 2.04) | 0.89 (0.21 – 3.78) | 0.49 (0.12 – 1.97) | 0.55 (0.11 – 2.65) | 0.45 (0.10 – 2.13) | 0.51 (1.53 – 1.73) | 0.42 (0.10 – 1.73) | - |

Supplemental Figure 3. Hazard Ratios with 95% Confidence Interval for OS in the Random Effects Model

Supplemental Figure 4. CINeMA Analysis for OS.


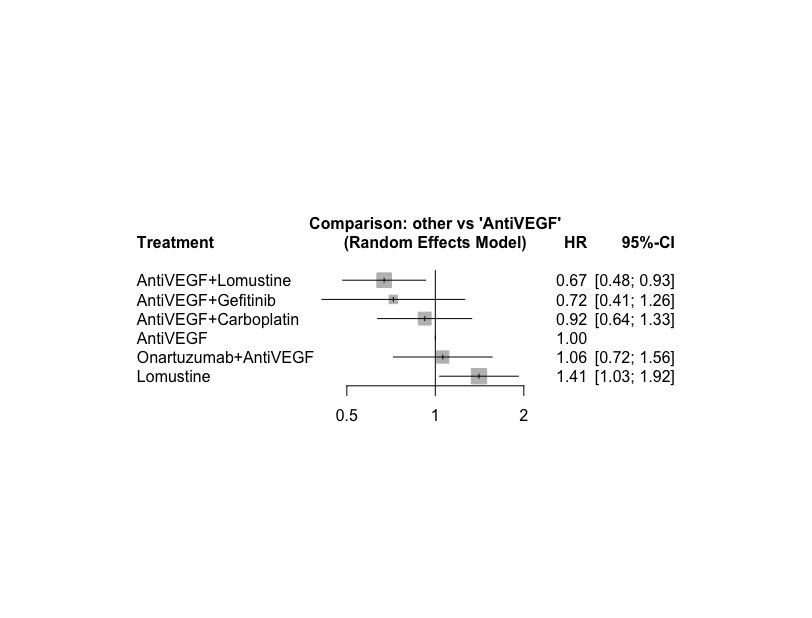


A

B


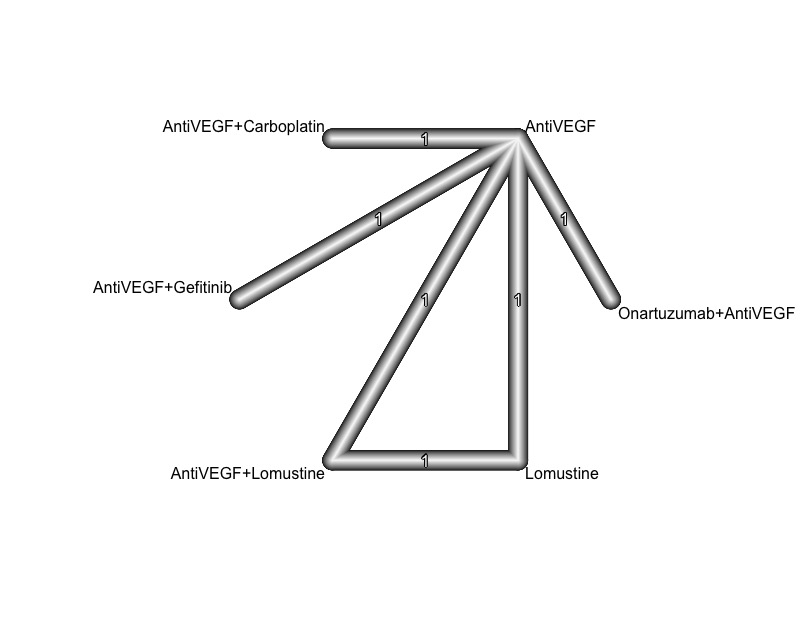


Supplemental Figure 5. Sensitivity analysis for PFS with A) forest plot and b) network node graph.


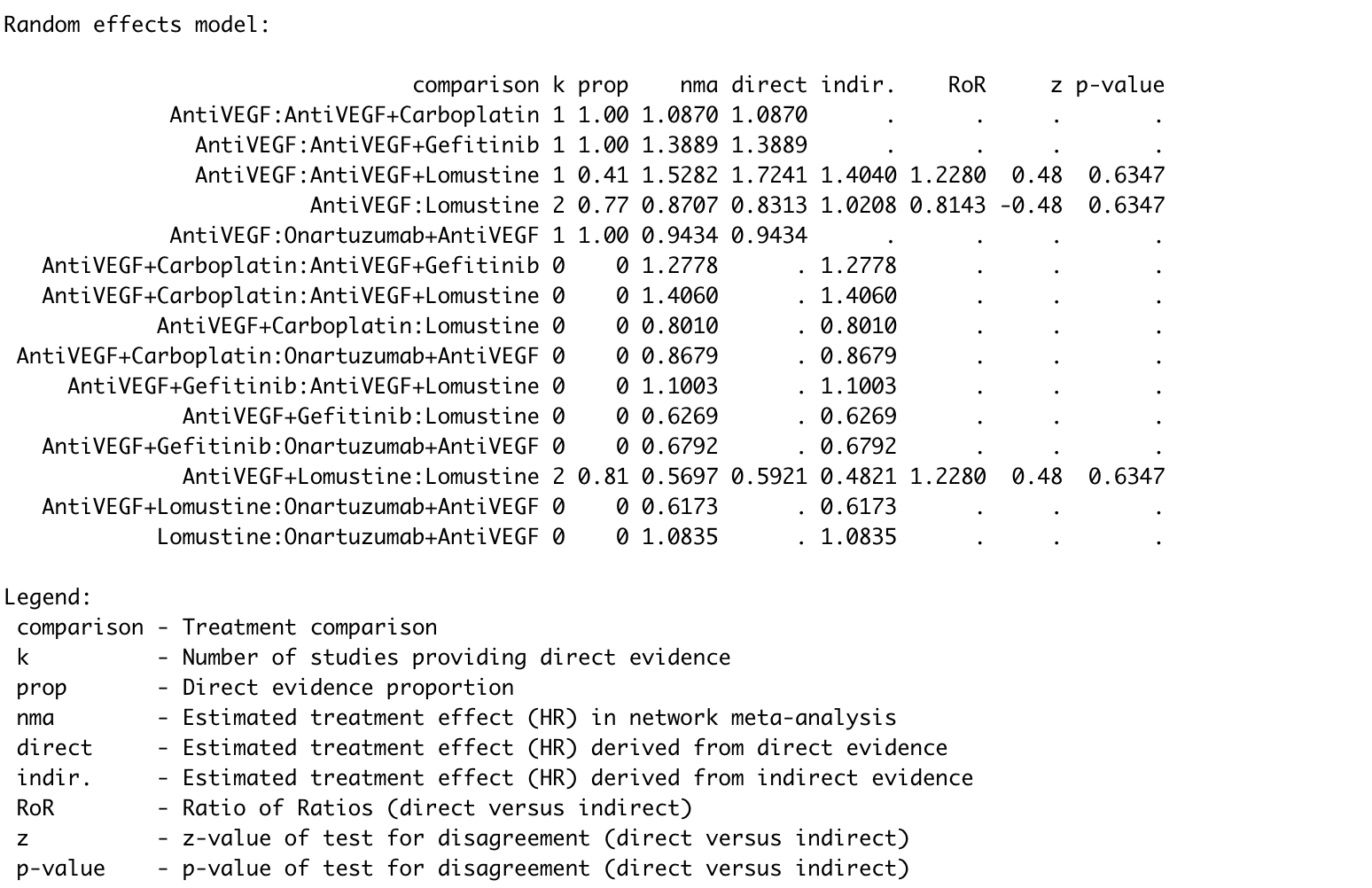


Supplemental Figure 6. Random effects model used in the calculation of PFS

| Treatment | AntiVEGF | AntiVEGF + Carboplatin | AntiVEGF + Gefitinib | AntiVEGF + Lomustine | Lomustine | Onartumzumab + AntiVEGF |
| --- | --- | --- | --- | --- | --- | --- |
| AntiVEGF | - | 1.09 (0.62-1.92) | 1.39 (0.68-2.82) | 1.53 (1.00 – 2.32) | 0.87 (0.61 – 1.24) | 0.94 (.53-.169) |
| AntiVEGF + Carboplatin | 0.92 (0.52-1.63) | - | 1.28 (0.51-3.17) | 1.41 (0.69-2.85) | 0.80 (0.41 – 1.57) | 0.87 (0.38 – 1.96) |
| AntiVEGF + Gefitinib | 0.72 (0.35-1.46) | 0.78 (0.32-1.94) | - | 1.10 (0.483-2.50) | 0.63 (0.28 – 1.38) | 0.68 (0.27 – 1.70) |
| AntiVEGF + Lomustine | 0.65 (0.43-0.99) | 0.71 (0.35-1.44) | 0.91 (0.40-2.07) | - | 0.57 (0.41 – 0.79) | 0.62 (0.30 – 1.26) |
| Lomustine | 1.15 (0.81-1.64) | 1.25 (0.64-2.44) | 1.60 (0.72-3.52) | 1.76 (1.26-2.44) | - | 1.08 (0.55 – 2.1) |
| Onartumzumab + AntiVEGF | 1.06 (0.59-1.90) | 1.15 (0.51-2.60) | 1.47 (0.59-3.68) | 1.62(0.79 – 3.31) | 0.92 (.47 – 1.82) | - |

Supplemental Figure 7. Hazard Ratios and 95% confidence interval in the Random Effects Model for PFS.

Supplemental Figure 8. CINeMA Analysis for PFS

| Study | Treatment | No. Patients | Average Adverse Events Per Patient | | |
| --- | --- | --- | --- | --- | --- |
|  |  |  | Grade 3 | Grade 4 | Grade5 |
| Batchelor (2012) | Lomustine | 65 | 0.6 | | - |
|  | Cediranib | 129 | 0.6 | | - |
|  | Cediranib/Lomustine | 131 | 0.75 | | - |
| Brandes (2016) | Galunisertib/Lomustine | 79 | .51 | | - |
|  | Galunisertib | 39 | .667 | | - |
|  | Lomustine | 40 | .98 | | - |
| Brown (2016) | Cediranib | 19 | 1.42 | | - |
|  | Cediranib/Gefitinib | 19 | 2.53 | | - |
| Cloughesy (2017) | Onratuzumab/Bevacizumab | 91 | .274 | | 2 |
|  | Bevacizumab | 53 | .434 | | 0.01 |
| Field (2015) | Bevacizumab | 62 | .242 | | |
|  | Bevacizumab/Carboplatin | 60 | 0.30 | | |
| Lombardi (2019) | Lomustine | 60 | 0.45 | - | - |
|  | Regorafenib | 59 | .58 | 0.02 | - |
| Stupp (2012) | TTF alone | 120 | 1.32 | | - |
|  | "Active chemo" | 117 | 0.85 | | - |
| Wick (2017) | Lomustine | 149 | 0.38 | | |
|  | Bevacizumab/Lomustine | 288 | 0.63 | | |

Supplemental Table 1. Summary of AE’s and frequency
